# Designing phage cocktails to combat the emergence of bacteriophage-resistant mutants in multidrug-resistant *Klebsiella pneumoniae*

**DOI:** 10.1128/spectrum.01258-23

**Published:** 2023-11-29

**Authors:** Seongjun Yoo, Kang-Mu Lee, Nayoung Kim, Thao Nguyen Vu, Ricardo Abadie, Dongeun Yong

**Affiliations:** 1 Department of Laboratory Medicine and Research Institute of Bacterial Resistance, Yonsei University College of Medicine, Seoul, South Korea; 2 Department of Laboratory Medicine, Graduate School of Medical Science, Brain Korea 21 Project, Yonsei University College of Medicine, Seoul, South Korea; 3 MicrobiotiX Co., LTD, Seoul, South Korea; University of Pittsburgh School of Medicine, Pittsburgh, Pennsylvania, USA

**Keywords:** bacteriophage, phage cocktail, phage resistance, *Klebsiella pneumoniae*

## Abstract

Phage therapy, an emerging strategy in the fight against recalcitrant bacterial infections, encounters a significant hurdle posed by the development of phage-resistant bacterial strains. To address this concern, we suggest a novel phage cocktail. By subjecting bacterial strains to a gradual selection process, we isolated and identified three novel phages augmenting one previously isolated phage. This diverse phage set formed the basis of a phage cocktail, displaying enhanced efficacy in curtailing the emergence of phage resistance, particularly evident in the notable stability achieved by three- and four-phage combinations. Our investigation further illuminated distinctive attributes exhibited by phage-resistant mutants: a concomitant reduction in growth rate and virulence offset by a noteworthy escalation in biofilm formation. The therapeutic modality underpinning our approach encompasses both the direct lethality of phages toward bacteria and the intricate landscape of bacterial genetic adaptations. Of intrigue was our observation that bacterial strains previously impervious to a given phage could regain susceptibility following resistance development against an unrelated phage. This phenomenon demonstrates the potential of phage cocktails as an efficacious countermeasure. Consequently, we suggest an innovative approach involving the design of phage cocktails that selectively encompass phages retaining susceptibility to bacteria that have developed resistance to other phages within the cocktail. Furthermore, we advocate for the comprehensive profiling of phage host ranges targeting both resistant strains and wild-type counterparts. The compilation of such intricate host-range data holds the promise of establishing a repository of diverse phages, meticulously tailored for refined and personalized phage therapy interventions.

**IMPORTANCE:**

In this study, we aimed to design a novel and effective bacteriophage cocktail that can target both wild-type bacteria and phage-resistant mutants. To achieve this goal, we isolated four phages (U2874, phi_KPN_H2, phi_KPN_S3, and phi_KPN_HS3) that recognized different bacterial surface molecules using phage-resistant bacteria. We constructed three phage cocktails and tested their phage resistance-suppressing ability against multidrug-resistant Klebsiella pneumoniae. We argue that the phage cocktail that induces resensitization of phage susceptibility exhibited superior phage resistance-suppressing ability. Moreover, we observed trade-off effects that manifested progressively in phage-resistant bacteria. We hypothesize that such trade-off effects can augment therapeutic efficacy. We also recommend collating phage host range data against phage-resistant mutants in addition to wild-type bacteria when establishing phage banks to improve the efficiency of phage therapy. Our study underscores the importance of phage host range data in constructing effective phage cocktails for clinical use.

## INTRODUCTION

The emergence of multidrug-resistant (MDR) bacteria poses an enormous threat to global public health (1, 2). We have combatted MDR bacteria by developing novel antibiotics whenever new MDR bacteria emerge. However, this approach has been unsuccessful owing to a decrease in investment in the development of new antibiotics and the depletion of novel antibiotic candidates (3). Furthermore, antibiotic resistance rates among bacteria are increasing annually (4 – 6). The World Health Organization (WHO) declared that we are entering a post-antibiotic era where no antibiotics can treat MDR bacterial infections (7, 8).

ESKAPE (*Enterococcus faecium, Staphylococcus aureus, Klebsiella pneumonia, Acinetobacter baumannii, Pseudomonas aeruginosa,* and *Enterobacter species*) are the main MDR bacterial species (9). *Klebsiella pneumoniae* (*K. pneumoniae*) is a gram-negative bacterium that causes infectious diseases, including pneumonia, urinary tract infection (UTI), and sepsis. *K. pneumoniae* is expanding its antibiotic resistance to β-lactams and quinolones (10). Therefore, a new strategy is required to manage MDR *K. pneumoniae* infections.

Bacteriophage (phage) therapy is a promising strategy for treating MDR bacterial infections (11 – 13). However, bacteria can acquire phage resistance similar to resistance to chemical antibiotics; therefore, this problem must be considered when applying phage therapy (14). Several bacterial phage resistance mechanisms, including blocking phage adsorption, preventing and degrading phage nucleic acid entry (restriction-modification system, CRISPR/Cas system), and abortive infection systems, in which bacterial cells self-destruct before phage propagation, have been reported (15). Recently, a novel mechanism has revealed that bacteria express nucleotide modification proteins that convert their nucleotide pool, inhibit phage replication, and induce abortive infection cell death (16). Among these mechanisms, blocking phage adsorption is critical to phages as it inhibits the initial stage of infection. Phages recognize molecules on the bacterial cell surface and bind specifically; however, mutations in these molecules would lead to a lack of recognition by the phages, causing phage resistance (17).

Several methods, such as phage training, genetically engineered phages, and phage cocktails, have been developed to overcome phage resistance in bacteria (18 – 20). The phage cocktail is a mixture of different phages expected to suppress phage resistance and have a broader host range than a single phage. However, randomly mixed phage cocktails exhibit fluctuating bactericidal effects because of the interaction between cocktail component phages (21). Phages that recognize the same surface molecule can act as antagonists to each other and simultaneously fail to infect bacteria that harbor surface molecules with mutations. Furthermore, phages can block nucleic acid injection of genetically similar phages to inhibit competitor phage propagation, which is known as a superinfection-exclusive system (15).

Thus, selecting phages that recognize different surface molecules is essential for phage cocktail therapy (22). However, sorting phages that recognize different surface molecules requires considerable time, money, and labor. Previous studies have isolated phages that easily recognize different surface molecules using phage-resistant mutants (23, 24). If bacteria acquire phage resistance by adsorption blocking, phages isolated from phage-resistant bacteria induced by another phage are considered to recognize different surface molecules. Thus, we can readily isolate novel phages that recognize different surface molecules using phage-resistant mutant bacteria.

When bacteria undergo mutations to acquire resistance against phages, they often experience a trade-off effect, which leads to the acquisition of unfavorable characteristics. These characteristics encompass a range of traits, including a decreased growth rate, reduced virulence, and an increased susceptibility to antibiotics (25 – 27). The trade-off effect arises as the genetic changes necessary for phage resistance can impose costs on other aspects of the bacteria’s biology (26, 28, 29). Thus, we assumed that the progressive inducing resistance in bacteria can result in the cumulative accumulation of trade-off effects, ultimately placing their survival at a disadvantage position.

Based on this hypothesis, we propose the utilization of a phage cocktail consisting of phages that target bacteria exhibiting adsorption-blocking resistance to phages. This approach aims to suppress phage resistance while imposing a disadvantageous trade-off effect on bacteria. In addition, we present phage host range data against phage-resistant bacteria in our phage bank. We believe that this information can contribute to the development of additional phage cocktails capable of suppressing phage resistance, thereby facilitating the widespread application of phage therapy to combat phage-resistant bacteria.

## RESULTS

### Isolation of phage-resistant bacteria and novel phages

**Fig 1:**
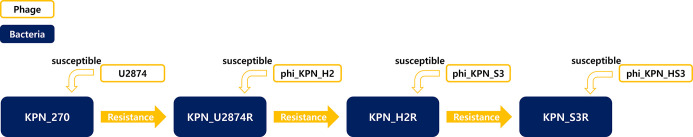
Workflow of novel phage isolation. We induced phage-resistant mutants and sequentially isolated a novel phage. KPN_270 is a wild-type bacterial strain. KPN_U2874R, KPN_H2R, and KPN_S3R are first-, second-, and third-stage phage-resistant mutants, respectively. U2874 is the first phage that was previously isolated. phi_KPN_H2, phi_KPN_S3, and phi_KPN_HS3 were, respectively, the second, third, and fourth phages that newly isolated.

In this study, we repeatedly induced phage-resistant mutant bacteria and isolated novel phages from various environmental samples (Fig. 1). The original bacterial strain, KPN_270 was isolated from the patient at the University Hospital (Severance, Seoul, Korea). We induced phage U2874 resistance in KPN_270 by co-culturing for 24 hours. We named the KPN_270 mutant, which exhibits resistance against phage U2874, as KPN_U2874_R. Subsequently, we isolated a novel phage, phi_KPN_H2 (OQ267593.1), from the Han River sample (N37°32′22″, E126°54′07″; Seoul, Korea) that is effective against KPN_U2874_R. We induced phi_KPN_H2-resistant mutant in KPN_U2874_R using the same method as mentioned before and named KPN_H2_R which exhibits the phi_KPN_H2 resistance. The novel phage phi_KPN_S3 (OQ267591.1) that was effective against KPN_H2_R was isolated from the Seo-Ho stream (N37°18′50″, E126°59′02″; Suwon, Korea). KPN_S3_R was induced from KPN_H2_R which exhibits resistance to phi_KPN_H2. Finally, the novel phage phi_KPN_HS3 (OQ267592.1) was isolated from hospital sewage (Severance, Seoul, Korea) that was effective against KPN_S3_R. In summary, we have isolated three phage-resistant mutants (KPN_U2874_R, KPN_H2_R, and KPN_S3_R) as well as three novel phages (phi_KPN_H2, phi_KPN_S3, and phi_KPN_HS3) each of which is effective against the corresponding phage-resistant mutants.

### Phage-resistant mechanism

**Fig 2:**
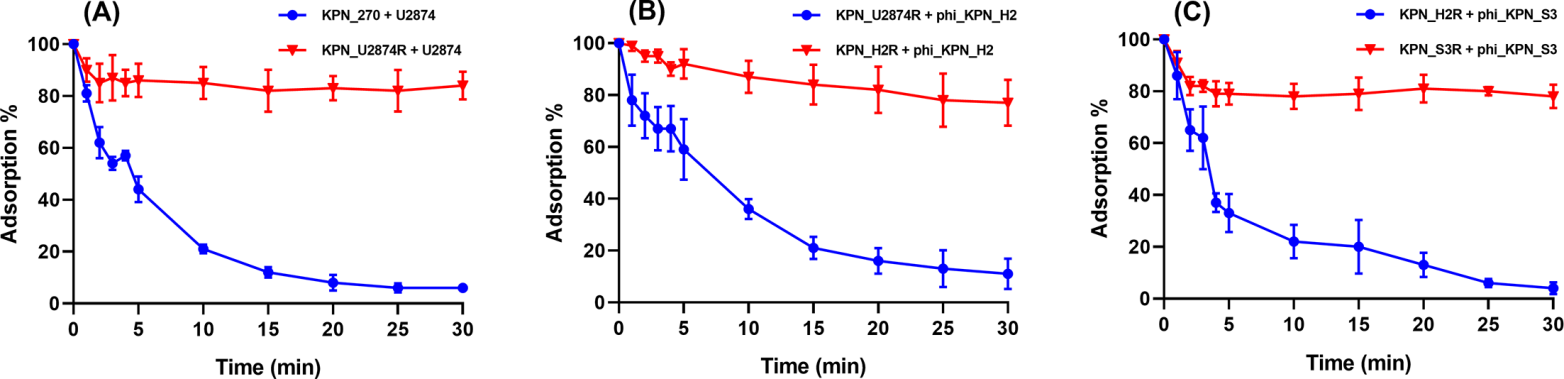
Results of adsorption tests depicting the phage-resistance mechanism. All the phage-resistant mutants exhibited reduced adsorption rates compared with that of the original host strain. Adsorption tests were performed in triplicate. (**A**) Bacteria KPN_270 and KPN_U2874R against phage U2874. (**B**) Bacteria KPN_U2874R and KPN_H2R against phi_KPN_H2. (**C**) Bacteria KPN_H2R and KPN_S3R against phi_S3

We conducted an adsorption assay to validate whether the inhibition of adsorption serves as a mechanism of phage resistance. Each phage was incubated with the host and phage-resistant mutant bacteria. For example, phage U2874 was incubated with the original host, KPN_270 as a control, and the resistant mutant, KPN_U2874R. After 30-minute incubation, all three phages exhibited over 90% adsorption rates (94%, 90%, and 96%, respectively) to their original host (Fig. 2). However, they showed an average of 20% adsorption rate (16%, 23%, and 22%, respectively) for their resistant mutants (Fig. 2). Based on these results, we have elucidated that the phage resistance mechanism is attachment inhibition.

### Phage receptor screening

To elucidate the genes conferring phage resistance, transposon random mutagenesis was conducted. Through mutagenesis, the genes associated with phage resistance were identified indirectly in the host bacteria (Table S1). As a result, KPN_270 displayed resistance when mutations occurred in the *wzi*, *wza*, and *galE* genes, indicating resistance to U2874. These genes are related to the synthesis of capsular polysaccharide (CPS) in *K. pneumoniae*, with U2874 being recognized as a CPS receptor (30).

KPN_U2874R exhibited resistance to phi_KPN_H2 upon mutations in the *fhuA* and *fhuC* genes. *FhuA* is a ferrichrome outer membrane transporter, and *fhuC* is an ATPase, thereby revealing that phi_KPN_H2 utilizes the membrane protein *fhuA* as its receptor (31).

KPN_H2R gained resistance to phi_KPN_S3 due to mutations in the *waaC*, *waaG*, and *galU* genes. These genes are related to lipopolysaccharide (LPS) core synthesis, elucidating that the receptor for phi_KPN_S3 is the LPS core (32).

Finally, KPN_S3R displayed resistance to phi_KPN_HS3 upon mutations in the *tonB* gene. Although *tonB* is not a direct receptor for the phage, being an inner membrane protein, it interacts with various outer membrane proteins (33). Therefore, it was deduced that the receptor for phi_KPN_HS3 is a tonB-dependent transporters (TBDTs), as it engages in interactions with multiple outer membrane proteins (34).

### Host range of phages

**Fig 3:**
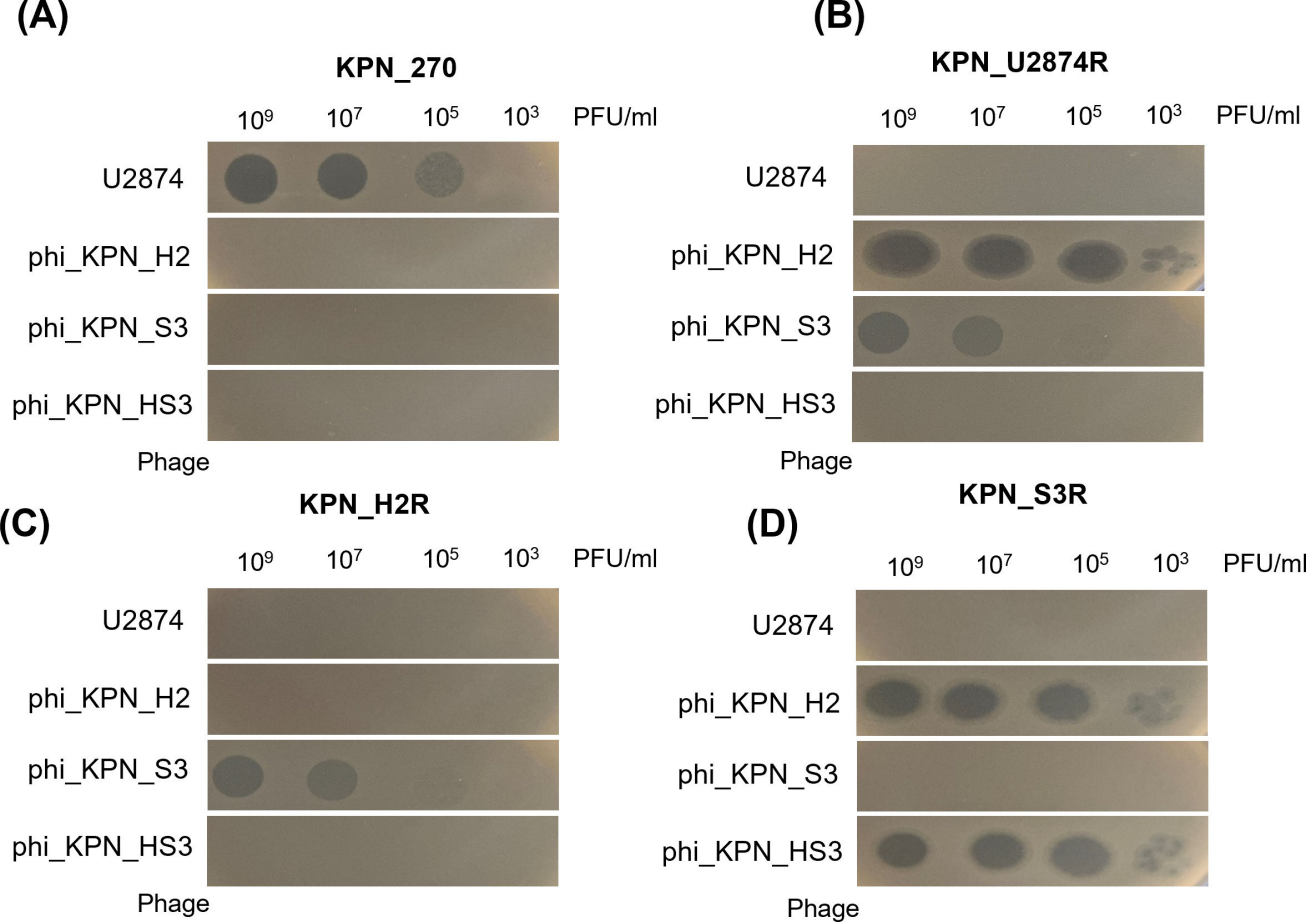
Phage host spectrums against four bacterial strains used in this study. Phage samples were serially diluted 100-fold, and 10 µL was spotted for each diluent.

We also performed spot tests to confirm the phage host range of each phage-resistant mutant and the phage (Fig. 3). Phage U2874 demonstrated efficacy against only KPN_270 (original host). phi_KPN_H2 exhibited efficacy against KPN_U2874R (the original host) and KPN_S3R. phi_KPN_S3 showed efficacy against KPN_U2874R and KPN_H2R (the original host). Lastly, phi_KPN_HS3 showed efficacy against only KPN_S3R (original host). Notably, phi_KPN_H2 and phi_KPN_S3 were effective against resistant mutants of each other.

### Physical and genetic features of phages

**Fig 4:**
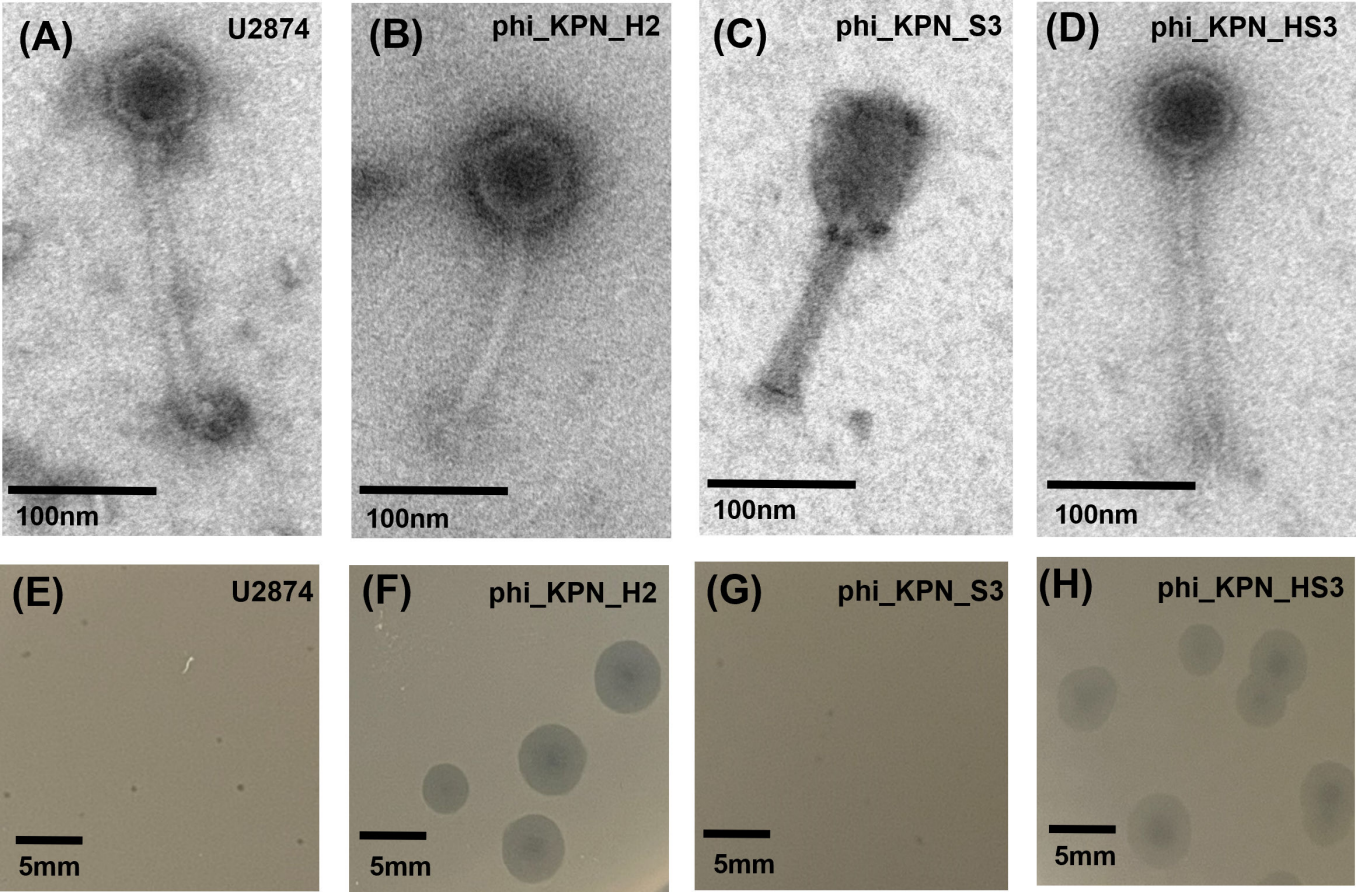
TEM images of phage U2874 (**A**), phi_KPN_H2 (**B**), phi_KPN_S3 (**C**), and phi_KPN_HS3 (**D**). Phage plaque morphologies of U2874 (**E**), phi_KPN_H2 (**F**), phi_KPN_S3 (**G**), and phi_KPN_HS3 (**H**). Black bars indicate the scale.

Transmission electron microscopy (TEM) images revealed that U2874, phi_KPN_H2, and phi_KPN_HS3 had icosahedral heads of 56 ± 2, 72 ± 2, and 59 ± 2 nm in diameter, and tails of 163 ± 4, 122 ± 3, and 199 ± 3 nm, respectively. Furthermore, phi_KPN_S3 had a prolate head morphology of 65 ± 2 nm in diameter, 72 ± 2 nm in head length, and a tail of 105 ± 2 nm (Fig. 4A through D; Table 1).

**TABLE 1:** Characteristics of phages

Characteristic	Phage			
	U2874	phi_KPN_H2	Phi_KPN_S3	phi_KPN_HS3
Head morphology	Icosahedral	Icosahedral	Prolate	Icosahedral
Head length (nm)	56 ± 2	72 ± 2	80 ± 2	59 ± 2
Head diameter (nm)	56 ± 2	72 ± 2	65 ± 2	59 ± 2
Tail length (nm)	163 ± 4	122 ± 3	105 ± 2	199 ± 3
Genome size (bp)	59,087	60,324	176,202	49,111
GC content (%)	56.3	45.62	41.87	50.9
CDSs	80	103	281	75
tRNA	0	1	1	0
Circular	Yes	Yes	Yes	Yes

The double-layer agar method was used to identify phage plaque morphology. Phage U2874 exhibited small and clear plaques approximately 1 mm in diameter. phi_KPN_H2, phi_KPN_S3, and phi_KPN_HS3 demonstrated plaques with clear centers and turbid edges. Among them, phi_KPN_H2 and phi_KPN_HS3 showed relatively large plaques, approximately 6 mm in diameter. By contrast, phi_KPN_S3 had relatively small plaques approximately 1 mm in diameter (Fig. 4E through H).

**Fig 5:**
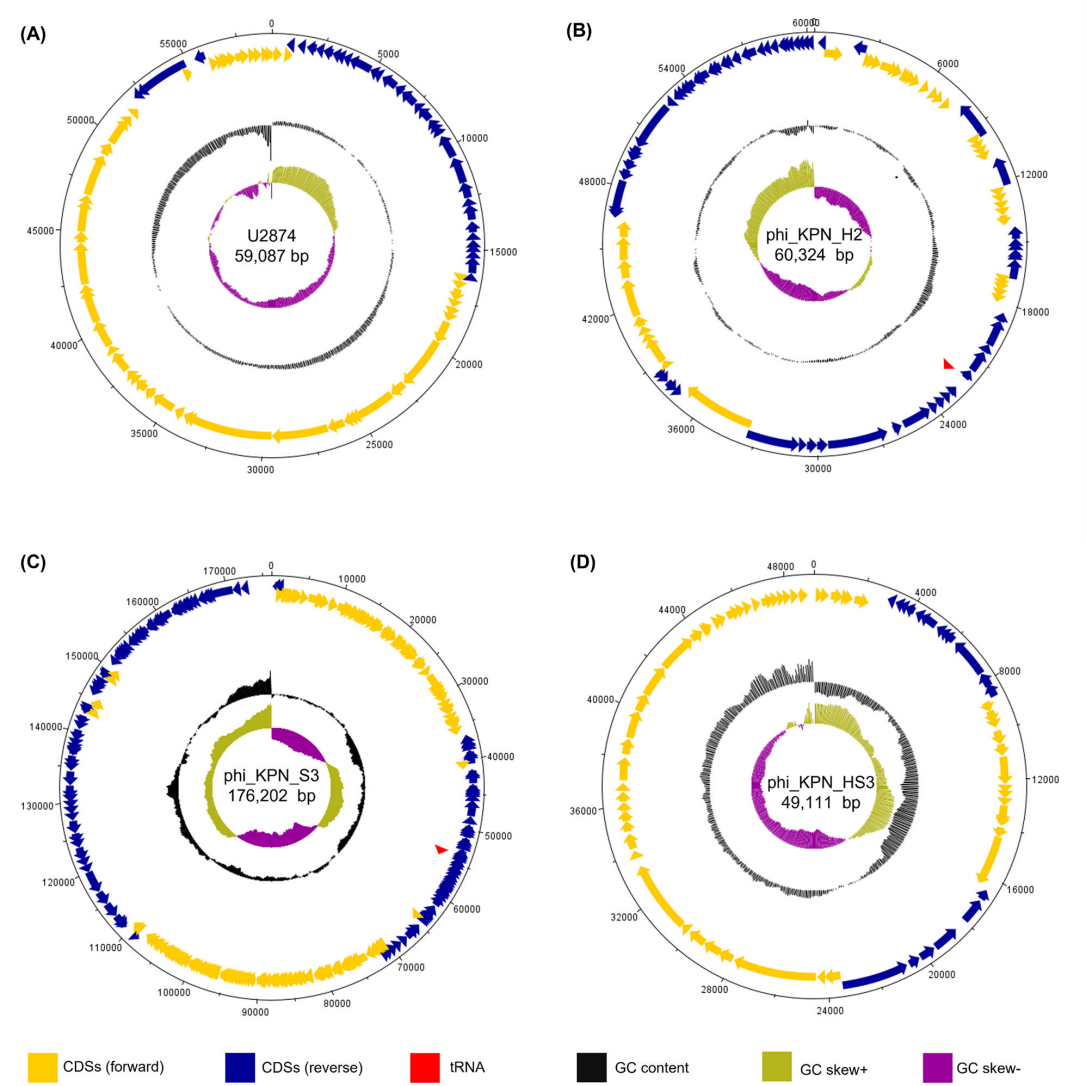
Circular map of (**A**) U2874, (**B**) phi_KPN_H2, (**C**) phi_KPN_S3, and (**D**) phi_KPN_HS3 genomes using DNAPlotter. Outer black lines represent the scales of genome sizes. Yellow arrows indicate forward CDSs and dark blue arrows indicate reverse CDSs. Arrows represent the 5′–3′ direction of each CDSs. Inner rings indicate GC content (black) and GC skew (+, olive color; −, purple). The whole genome sizes and names of phages are represented at the center of the circular map.

Whole-genome sequencing revealed that all four phages (U2874, phi_KPN_H2, phi_KPN_S3, and phi_KPN_HS3) have circular double-stranded DNA, and alignment results using Mauve (https://darlinglab.org/) and BLAST (data not provided) suggested that they lacked homologous sequences (35). The GC content of the four phages was diverse. Phages U2874, phi_KPN_H2, phi_KPN_S3, and phi_KPN_HS3 showed 56.3%, 45.62%, 41.87%, and 50.9% of GC content, respectively. Furthermore, the genome size was varied (59,087, 60,324, 176,202, and 49,111 bp, respectively). phi_KPN_S3 and phi_KPN_H2, the two phages with the largest genome size, encoded one tRNA gene and 103 and 281 CDSs, respectively. Phages U2874 and phi_KPN_HS3 did not encode tRNA genes but encoded 80 CDSs and 75 CDSs, respectively (Fig. 5; Table 1). The complete genomes of newly isolated phages (phi_KPN_H2, phi_KPN_S3, and phi_KPN_HS3) were deposited into the GenBank database under accession numbers OQ267591, OQ267592, and OQ267593.

### Suppression of phage resistance by phage cocktails

We conducted a lysis test to determine whether other phages within the cocktail can inhibit the growth of bacteria that have acquired phage resistance. The growth of the bacteria was evaluated by measuring the optical density (OD_600_ nm), and the observation of regrowth was considered as the emergence of resistant bacteria.

**Fig 6:**
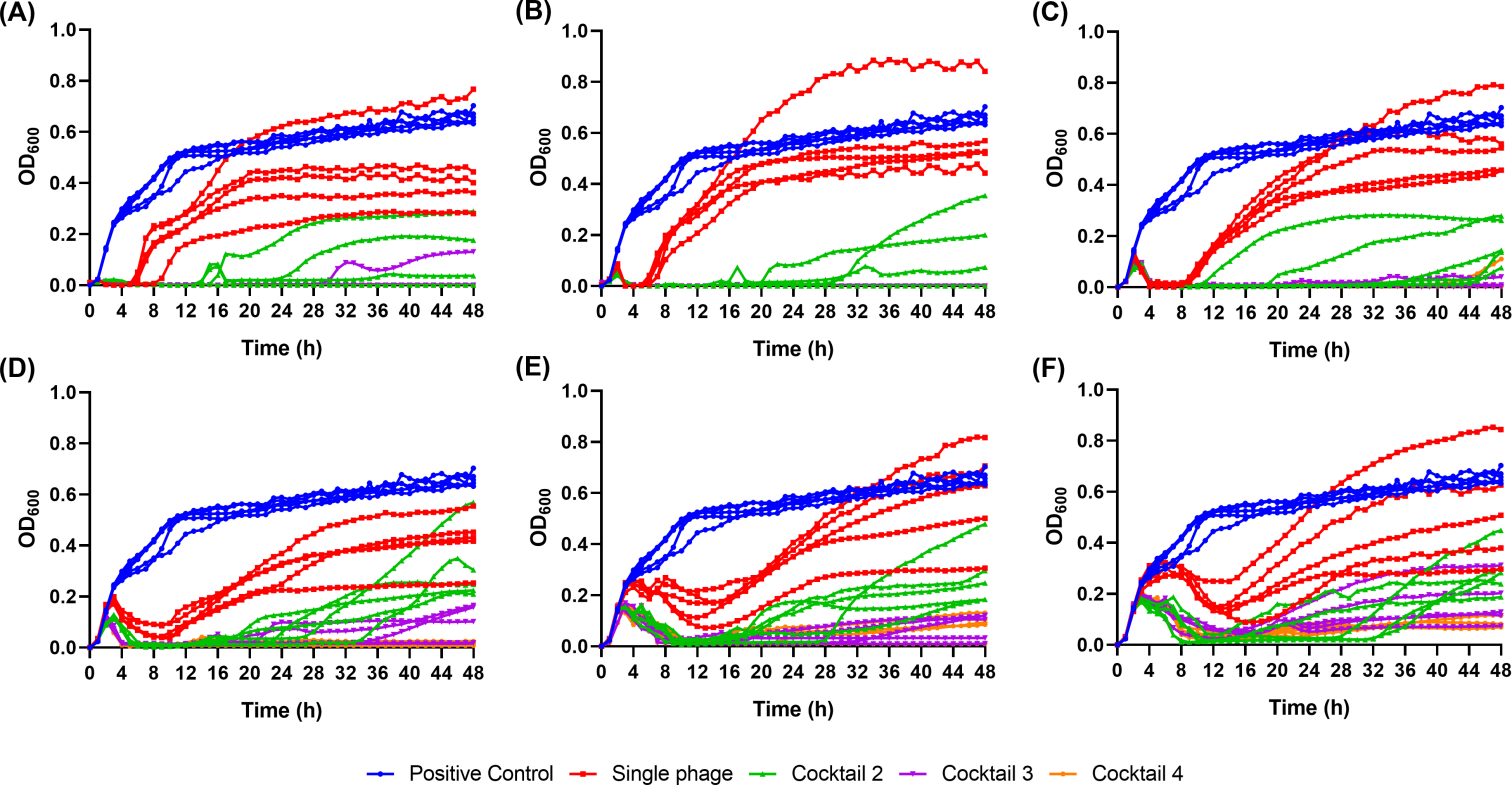
Lysis test results of single phage and phage cocktails. (**A**) MOI 10, (**B**) MOI 1, (**C**) MOI 0.1, (**D**) MOI 0.01, (**E**) MOI 0.001, and (**F**) MOI 0.0001. All the tests were performed in quintuplicate. (Single Phage; U2874, Cocktail 2; U2874 +phi_KPN_H2, Cocktail 3; U2874 +phi_KPN_H2 + phi_KPN_S3, Cocktail 4; U2874 +phi_KPN_H2 + phi_KPN_S3 + phi_KPN_HS3)

In the case of a single phage, continuous bacterial growth was observed even after regrowth was observed across all multiplicity of infections (MOIs) (Fig. 6). Furthermore, compared to the cocktail, the single phage exhibited an earlier onset of regrowth. Conversely, the cocktail effectively suppressed bacterial growth for a duration of 48 hours or delayed the emergence of resistant bacteria. In addition, growth suppression was observed following regrowth, implying that the growth of resistant bacteria was subsequently inhibited by other phages present in the cocktail. Moreover, we observed that cocktails 3 and 4 exhibited greater stability in inhibiting the emergence of phage-resistant bacteria compared to cocktail 2.

### Growth reduction in phage-resistant mutants

**Fig 7:**
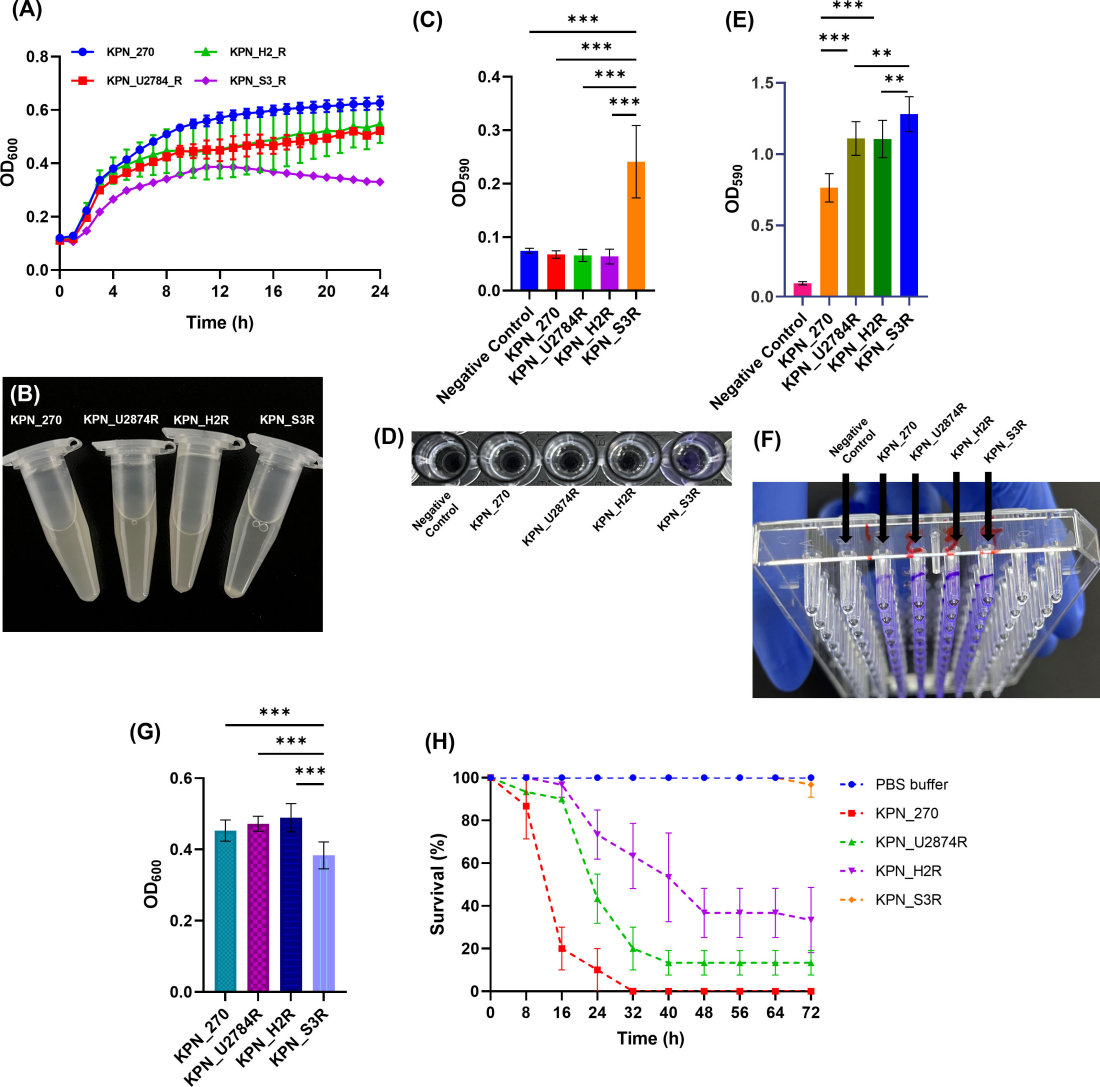
(**A**) The 24-hour growth curves of wild-type bacteria and phage-resistant mutants were measured at 600 nm wavelength. (**B**) Sedimentation of bacterial cells after 30 minutes of incubation with agitation was observed. (**C-D**) Biofilm formation on the bottom surface was quantified using stained crystal violet and measured at 590 nm. (**E-F**) Biofilm formation on lids was evaluated. Stained crystal violet was used to quantify the biofilm, and measurements were taken at 590 nm. (**G**) Bacterial growth in static culture was assessed by measuring optical density at the 24-hour time point, using a wavelength of 600 nm. (**H**) Survival rates of larvae infected with each bacterial strain were determined. One-way ANOVA followed by Tukey’s multiple comparisons test was used to compare the biofilm formation (***, *P* < 0.001; **, *P* = 0.007; ##, *P* = 0.005)

We observed bacterial growth over a 24-hour period (Fig. 7A). The results revealed that both KPN_H2R and KPN_S3R exhibited significant growth reduction compared to the wild-type KPN_270. Moreover, KPN_S3R displayed a more pronounced growth reduction than the phage-resistant mutants of the other two strains (KPN_U2873R and KPN_H2R).

### Increase in biofilm formation of phage-resistant mutants

We compared biofilm formation in our study. When subjected to static cultivation, KPN_S3R exhibited the characteristics of cell sedimentation (Fig. 7B). Given the potential impact of such traits on biofilm formation, we measured biofilm using two methods: the bottom and lid approaches. For the bottom biofilm, KPN_270, KPN_U2874R, and KPN_H2R showed no significant differences, while KPN_S3R displayed a noteworthy increase in biofilm formation compared to other strains (Fig. 7C and D). Similarly, in the lids biofilm, the KPN_S3R strain exhibited the highest biofilm formation. However, in contrast to the results at the bottom, both KPN_U2874R and KPN_H2R showed increased biofilm formation on lids compared to KPN_270 (Fig. E and F)

Furthermore, we verified whether the results of 24-hour static cultivation aligned with kinetic growth outcomes (Fig. 7G). After 24 hours of static cultivation, we measured the absorbance at 600 nm. The results showed that KPN_S3R displayed the most reduced growth compared to other strains, aligning to some extent with the kinetic growth results.

### Decrease in virulence of phage-resistant mutants in the *Galleria mellonella* infection model

We evaluated the virulence of bacterial strains that acquired phage resistance through a stepwise process using the Galleria mellonella infection model. The results demonstrated a gradual decrease in virulence as bacterial strains acquired resistance to phages (Fig. 7H). Larvae infected with KPN_270 experienced complete mortality within 32 hours of infection. For the second mutant, KPN_U2874R, the larvae displayed around 13% survival at 40 hours post-infection, which remained at 13% survival up to 72 hours. In the case of larvae infected with KPN_H2R, approximately 37% survived at 48 hours post-infection, decreasing to around 33% survival at 72 hours. Subsequently, larvae infected with the KPN_S3R mutant exhibited 100% survival up to 64 hours post-infection and around 97% survival after 72 hours.

### Changes in antibiotic susceptibility

We performed AST with GN4F Sensititre (Thermo Fisher, Waltham, MA, USA) and determined the antibiotic susceptibility according to the Clinical and Laboratory Standards Institute (CLSI) guidelines. The AST results indicated a minimal alteration in antibiotic susceptibility (Table 2). Comparing the wild type to all phage-resistant mutants, the minimum inhibitory concentration (MIC) decreased from 8 to 4 mg/mL for both tetracycline (TET) and minocycline (MIN).

**TABLE 2:** Antibiotic susceptibility and MIC of bacteria^a^

Bacterial strains	Antibiotics																							
	AMI	P/T4	TGC	TIM2	LEVO	NIT	TET	DOR	MIN	ETP	SXT	IMI	PIP	MERO	GEN	FAZ	TOB	TAZ	A/S2	AZT	AMP	FEP	CIP	AXO
KPN 270	8 S	128/4 R	1 S	64/2 R	8 R	64 R	8 I	4 R	8 I	8 R	4/76 R	8 R	64 R	8 R	2 S	16 R	2 S	16 R	16/8 R	16 R	16 R	32 R	2 R	32 R
KPN U2874R	8 S	128/4 R	1 S	64/2 R	4 S	64 R	4 S	4 R	4 S	8 R	4/76 R	8 R	64 R	8 R	2 S	16 R	2 S	16 R	16/8 R	16 R	16 R	32 R	2 R	32 R
KPN H2R	8 S	128/4 R	1 S	64/2 R	4 S	64 R	4 S	4 R	4 S	8 R	4/76 R	8 R	64 R	8 R	2 S	16 R	2 S	16 R	16/8 R	16 R	16 R	32 R	2 R	32 R
KPN S3R	8 S	128/4 R	1 S	64/2 R	4 S	64 R	4 S	4 R	4 S	8 R	4/76 R	8 R	64 R	8 R	2 S	16 R	2 S	16 R	16/8 R	16 R	16 R	32 R	2 R	32 R

^a^ AMI: amikacin, P/T4: piperacillin/tazobactam constant 4, TGC: tigecycline, TIM2: ticarcillin/clavulanic acid constant 2, LEVO: levofloxacin, NIT: nitrofurantoin, TET: tetracycline, DOR: doripenem, MIN: minocycline, PIP: piperacillin, MERO: meropenem, GEN: gentamycin, FAZ: cefazolin, TOB: tobramycin, TAZ: ceftazidime, A/S2: ampicillin/sulbactam, AZT: aztreonam, AMP: ampicillin, FEP: cefepime, CIP: ciprofloxacin, AXO: ceftriaxone; S: susceptible, I: intermediate, R: resistance; number: minimal inhibitory concentration (MIC).

### Observation of bacterial cell morphology

**Fig 8:**
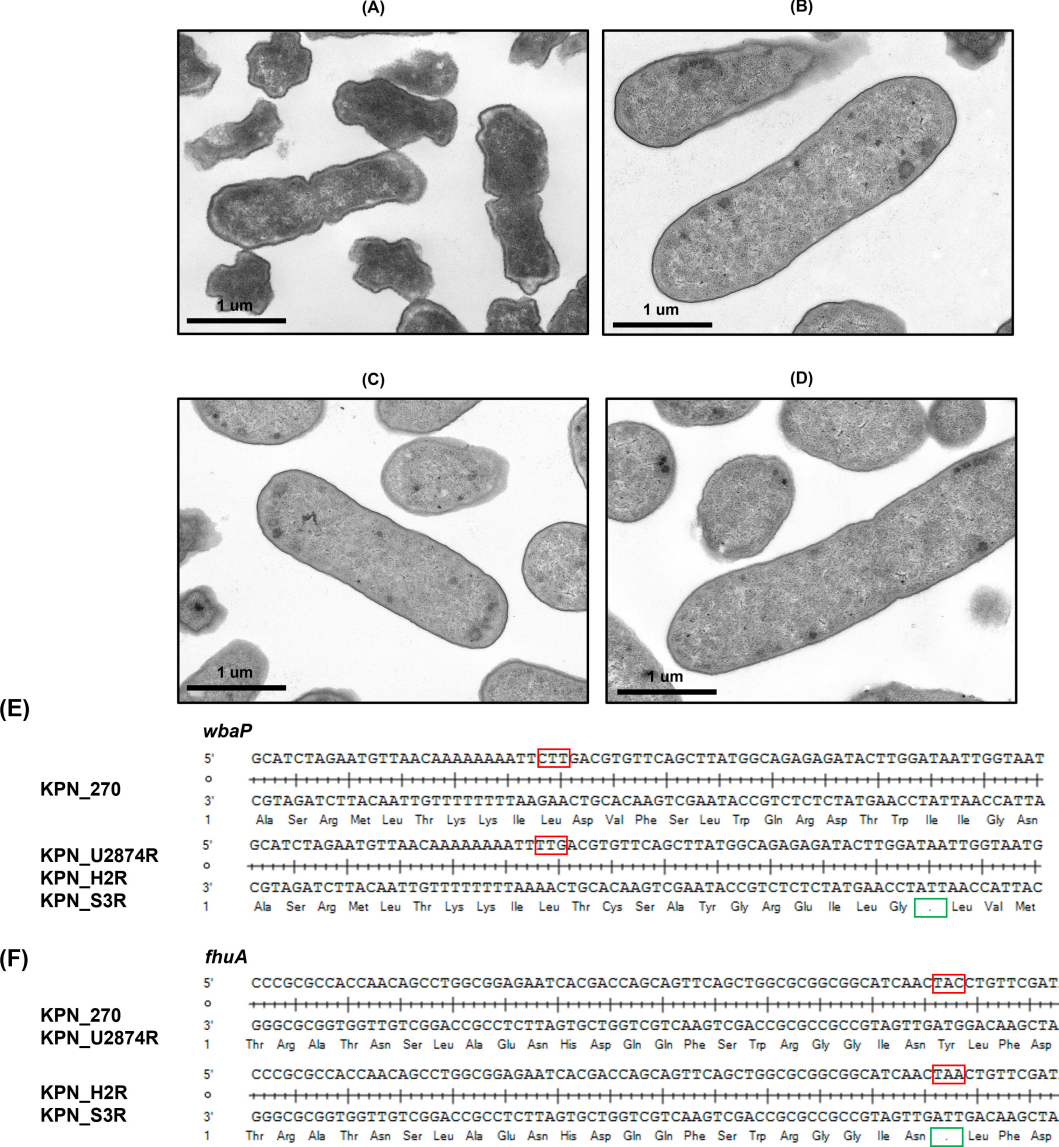
TEM images of bacterial cell morphology of KPN_270 (**A**), KPN_U2874R (**B**), KPN_H2R (**C**), and KPN_S3R (**D**). Black bars indicate the scale. Nonsense mutation of gene *wbaP* (**E**) and *fhuA* (**F**). Red boxes indicate codons where the mutation occurred. Green boxes indicated the premature stop codon.

We conducted observations of bacterial cell morphology using TEM. The TEM imaging results revealed a reduction in extracellular matrix (ECM) among the phage-resistant bacteria (KPN_U2874R, KPN_H2R, and KPN_S3R) when compared to KPN_270 (Fig. 8A through D). In addition, a tendency toward cell elongation was observed, with KPN_S3R displaying the most prominent elongation. This phenomenon can be attributed to the loss of ECM (36).

### Genetic mutation of phage-resistant mutants

We performed variant calling on the bacterial genome using KPN_270 as the reference (Macrogen Seoul, Korea). The results of variant calling revealed mutations in several genes among the phage-resistant strains (Table 3). KPN_U2874 exhibited mutations in the following genes: *bla_1*, *glpR*, *ltnD*, *otnK*, *tktA*, *yicJ*, *tuf1*, *syrM1*, *dgcP*, and *wbaP* (37 – 45). KPN_H2R acquired a mutation in the *fhuA* gene alongside those inherited from KPN_U2874R, while KPN_S3R acquired mutations in the *galU*, *helD*, and *hcnB* genes in addition to those originating from KPN_H2R mutations (31, 46 – 48). Among these mutations, missense mutations were observed in *bla_1*, *syrM1*, *dgcP*, and *galU* genes, nonsense mutations in *wbaP* and *fhuA* genes, and frameshift mutations in *otnK* and *helD* genes (Fig. 8E and F). The remaining genes exhibited silent mutations.

**TABLE 3:** List of mutation genes of phage-resistant mutants

Bacterial strain	Gene	Function	Mutation
KPN_U2874R	*bla1*	Beta-lactam resistance (37)	Missense
	*glpR*	Glycerol metabolism (38)	Silent
	*ltnD*	L-threonate catabolism (39)	Silent
	*otnK*	Carbohydrate metabolism (39)	Frameshift
	*tktA*	Carbohydrate metabolism (40)	Silent
	*yicJ*	Carbohydrate metabolism (41)	Silent
	*tuf1*	Elongation factor (43)	Silent
	*syrM*	Transcription factor (44)	Missense
	*dgcP*	c-di-GMP synthesis (45)	Missense
	*wbaP*	CPS synthesis (46)	Nonsense
KPN_H2R	*fhuA*	Iron transport (31)	Nonsense
KPN_S3R	*galU*	LPS synthesis (46)	Missense
	*helD*	DNA repair (47)	Frameshift
	*hcnB*	Hydrogen cyanide biosynthesis (48)	Silent

## DISCUSSION

Phage therapy is gaining attention as a novel paradigm for treating multidrug-resistant bacterial infections. However, challenges such as the emergence of phage-resistant bacteria pose significant hurdles that must be overcome for phage therapy to be widely adopted. Several studies have reported efforts to mitigate the emergence of phage-resistant bacteria by combining phages into cocktails. In the approach of Ling Yu et al., one type of phage that infects wild-type bacteria was used to induce phage resistance, resulting in the isolation of two types of phage-resistant bacteria. A new phage was isolated for each type of phage-resistant bacteria to formulate a phage cocktail. Similarly, in the study of Shayla Hesse et al., two types of phages that infect wild-type bacteria were used, inducing phage resistance with one type and isolating new phages capable of infecting phage-resistant bacteria to create a phage cocktail. The study of Chengcheng Li et al. also employed a similar strategy, inducing phage resistance with one type of phage that infects wild-type bacteria and isolating a new phage capable of infecting phage-resistant bacteria to formulate a phage cocktail.

In our study, we induced phage resistance using one type of phage that infects wild-type bacteria, and this process was repeated three times to isolate three different phages capable of infecting phage-resistant bacteria (Fig. 1). Phage cocktails were created using this approach. Our research involved analyzing the newly isolated phages and confirming the suppression of the emergence of phage-resistant bacteria through the phage cocktails produced using the described methodology. In addition, we elucidated the characteristics and genetic mutations that occur as bacteria progressively acquire phage resistance.

We observed the sensitization and subsequent resensitization of newly isolated phages (Fig. 3). Initially, the novel phages did not exhibit efficacy against the wild-type bacteria, KPN_270. However, they acquired efficacy following the development of phage resistance by the wild-type bacteria. Furthermore, we noted a resensitization phenomenon after the bacteria acquiring phage resistance. In particular, phi_KPN_H2 demonstrated an inability to infect the phage-resistant strain, KPN_H2R. However, this phage successfully infected the phage-resistant strain, KPN_S3R, which had undergone mutation in response to phi_KPN_S3. Therefore, it can be inferred that the mutation induced by phi_KPN_S3 confers susceptibility to phi_KPN_H2. Sensitization can be explained in relation to phage receptors and bacterial phage resistance mutations. In our study, using transposon random mutagenesis, we identified the receptors for the phages U2874, phi_KPN_H2, phi_KPN_S3, and phi_KPN_HS3 as capsule, *fhuA*, LPS core, and TBDTs, respectively (Table S1). Only U2874 could infect KPN_270, indicating its ability to recognize the capsule of KPN_270. The receptors for the other three phages (*fhuA*, LPS core, and TBDTs) seemed to be concealed by the capsule, preventing the phages from accessing their receptors and infecting the bacteria (49).

The mutant strain KPN_U2874R, which acquired resistance to U2874 in KPN_270, could be infected by phi_KPN_H2 and phi_KPN_S3. The reason behind this is that the acquisition of resistance in KPN_270 to U2874 resulted in a disruption in the capsule, enabling access to the receptors, *fhuA*, and LPS core, which are receptors for phi_KPN_H2 and phi_KPN_S3. The capsule disruption in KPN_270 was confirmed through sequencing and TEM (Fig. 8A through E). A nonsense mutation was identified in the *wbaP* gene within the capsule gene cluster (LT174595.1) of KPN_270 (Table 3; Fig. 8E). The *wbaP* gene is involved in the assembly of polysaccharide units during capsule biosynthesis (30, 45). Thus, the missense mutation in the *wbaP* gene prevented the assembly of polysaccharide units, leading to capsule loss. In TEM images, the wild-type KPN_270 displayed a dark and uneven appearance in the extracellular region. Conversely, bacteria derived from KPN_U2874R, including KPN_U2874R itself, exhibited a relatively bright and smooth cell morphology (Fig. 8A through D).

For KPN_H2R, it exhibited susceptibility only to phi_KPN_S3 (Fig. 2C). Whole-genome sequencing (WGS) results revealed that KPN_H2R had a nonsense mutation in the *fhuA* gene (Table 3; Fig. 8F). This mutation in the *fhuA* gene explains how KPN_H2R acquired resistance to phi_KPN_H2, as *fhuA* is the receptor for phi_KPN_H2. Interestingly, the receptor for phi_KPN_S3 is the LPS core, and the *fhuA* mutation in KPN_H2R does not seem to affect the LPS core structure, allowing phi_KPN_S3 to still recognize the altered LPS core and maintain susceptibility in KPN_H2R.

In KPN_S3R, a missense mutation was observed in the *galU* gene (Table 3). The *galU* gene plays a crucial role in synthesizing UDP-galactose, an essential precursor for LPS biosynthesis (46). Furthermore, some studies suggest that mutations in *galU* can lead to LPS core defects (22, 46, 50). The *galU* mutation in KPN_S3R is consistent with its resistance to phi_KPN_S3. However, KPN_S3R retained susceptibility to phi_KPN_H2 and phi_KPN_HS3, with a notable resensitization of phi_KPN_H2 (Fig. 2D). Both phi_KPN_H2 and phi_KPN_HS3 have receptors that involve the *fhuA* and TBDTs. These outer membrane proteins, along with LPS, contribute to the stability of the bacterial outer membrane (51). For phi_KPN_HS3, it is hypothesized that the LPS core concealing the TBDTs was disrupted due to LPS core defects, leading to sensitization. However, further research is needed to verify this hypothesis.

Like phi_KPN_H2, phi_KPN_S3 also exhibited susceptibility to KPN_U2874R. To explore the phage host range, we isolated KPN_U2874R_S3R, a derivative of KPN_U2874R that acquired resistance to phi_KPN_S3. The results showed that there was no change in susceptibility to phi_KPN_H2, but phi_KPN_HS3 displayed sensitization in KPN_U2874R_S3R, similar to KPN_S3R’s range (Fig. S1). In conclusion, phi_KPN_H2 and phi_KPN_S3 did not influence each other’s susceptibility, and upon acquiring resistance to phi_KPN_S3, phi_KPN_HS3 showed sensitization. The resensitization of phi_KPN_H2 and sensitization of phi_KPN_HS3 might be associated with structural changes in bacterial cell architecture due to LPS core defects, although further research is needed to fully understand these phenomena.

In this study, we elucidated the trade-off phenomenon that occurs as bacteria acquire resistance to phages in a stepwise manner. We observed reductions in growth and virulence, as well as an increase in biofilm formation (Fig. 7). These trade-off effects resulting from the acquisition of phage resistance have been extensively reported in numerous studies (25 – 28, 45). The results obtained in this study align significantly with previously reported findings. The phage-resistant strains in this study exhibited deficiencies in both CPS and LPS. LPS and CPS are crucial molecules on the bacterial surface that play substantial roles in bacterial cell stability and virulence (45, 51 – 53). Notably, the strain KPN_S3R, which had deficiencies in both CPS and LPS, displayed considerable growth reduction and decreased virulence compared to strains with only CPS defects (Fig. 7A and H). Moreover, the KPN_S3R strain showed cell sedimentation and increased biofilm formation (Fig. 7B through F). Many studies have indicated that biofilm formation increases when either LPS or CPS is deficient (54 – 57). In addition, research suggests that interactions between bacterial cells through fimbriae are enhanced when LPS or CPS is deficient, leading to increased aggregation (55, 58).

Theoretically, if the cocktail designed in this study is applied for the treatment of multidrug-resistant bacteria, one can anticipate not only direct bacterial lysis due to the cocktail but also indirect therapeutic effects resulting from trade-off phenomena. However, since biofilm is also a significant virulence factor (59, 60), further research is necessary to investigate the indirect therapeutic effects related to biofilm increase when applying the cocktail.

### Conclusion

Through this study, we have illuminated the potential of a novel phage cocktail design. When bacteria acquire resistance to one phage, there is a characteristic where other phages become sensitized to the resistant strain, resulting in the suppression of resistant strain emergence. Particularly noteworthy is the phenomenon of resensitization, where bacteria that have acquired resistance to one phage regain susceptibility to previously resistant phages when acquiring additional resistance to different phages. This resensitization phenomenon among phage cocktails suggests the potential to achieve the effects of multiple phages using a smaller number of phages, thereby inhibiting the emergence of phage-resistant bacteria.

Furthermore, stepwise acquisition of phage resistance by the bacteria led to a trade-off effect, resulting in decreased growth rates and reduced virulence. Based on these findings, in theory, applying the cocktail designed in this study for the treatment of multidrug-resistant bacteria could yield not only direct bacteriolytic effects from the phages but also indirect therapeutic effects due to the trade-off phenomenon.

In the global effort to combat multidrug-resistant bacteria, some institutions have established phage banks to compile phage cocktails. However, to the best of our knowledge, phage banks only contain phage host range data against host bacteria and not phage-resistant mutants. Including data on phage host range against phage-resistant mutants in these banks can facilitate the rapid development of phage resistance-suppressing cocktails similar to those explored in this study. Therefore, we recommend that phage host range data against phage-resistant mutants should be collated alongside wild-type bacteria when establishing phage banks.

## MATERIALS AND METHODS

### Isolation of phage-resistant mutant bacteria

We induced phage-resistant mutant bacteria by co-culturing them with bacteria and phages. The workflow is displayed in Fig. 1. We mixed Luria-Bertani broth (LB; BD, Sparks, MD, USA), overnight bacterial culture (~10^7^ CFU/mL), and phage (~10^6^ PFU/mL) diluent 100:1:1 (vol/vol) and incubated for 16–18 hours at 37°C with agitation at 180 rpm. The mixture was then diluted and spread on Mueller Hinton Agar (MHA; Asanpharm; Hwaseong, Korea) for bacterial colony isolation. We repeated colony isolation until a single colony appeared (at least three times) and confirmed phage resistance by spot testing as previously described (61). Subsequently, we stored the phage-resistant mutant bacteria in a 2:1:1 (vol/vol) mixture of LB broth, glycerol (VWR Life Science, Radnor, PA, USA), and distilled water at −70°C (62).

### Phage-resistant confirmation

We confirmed phage resistance by spot testing as previously described and confirmed the resistance mechanism by adsorption testing (17, 23). The adsorption test was performed as previously described with some modifications (61). We added the phage solution (~10^3^ PFU/mL) to the bacterial inoculum (~10^6^ CFU/mL). Phage samples were collected at 0, 1, 2, 3, 4, 5, 10, 15, 20, 25, and 30 minutes after co-culture and filtered with a 0.22-µm syringe filter (Sartorius, Göttingen, Germany) to isolate the phage. Using the double-layered agar method described previously, we measured phage titers (61). In this adsorption test, we tested two bacterial strains (original host and phage-resistant mutant) per phage and compared the two results to determine whether the phage resistance mechanism was blocking adsorption. In addition, we used the original host for measuring phage titers, and all tests were performed in triplicate.

### Novel phage isolation, purification, and morphology characterization

We isolated a novel phage using the 2 × LB broth method previously described, with minor modifications (63). Briefly, we mixed the 2 × LB broth, environment sample, and target bacterial inoculum (~10^7^ CFU/mL) 50:50:1 (vol/vol). And incubated for 16–18 hours at 37°C with agitation at 180 rpm. After incubation, the sample was centrifuged at 10,000× *g* for 10 minutes at 4°C and filtered using a 0.22-µm syringe filter. We used the double-layered agar method to check for phage plaques. We selected a single plaque using an inoculation loop and repeated this process until a single plaque was confirmed (at least three times).

For phage sample storage and *in vitro* use, the polyethylene glycol (PEG) precipitation method was used as previously described (61). Briefly, we added PEG (Sigma-Aldrich, St. Louis, MO, USA) 1% (vol/wt) and agitated until the PEG dissolved completely. The samples were then incubated for 16–18 hours at 4°C. After incubation, the samples were centrifuged at 24,000× *g* for 1 hour at 4°C. The supernatant was discarded, and the pellet was washed with SM buffer (100 mM NaCl, 8 mM MgSO_4_∙7H_2_O, 50 nM Tris-HCl; pH 7.5). Phage samples were stored at 4°C.

The phage morphology was identified using TEM. TEM was performed as previously described with minor modifications (61). The purified phage sample (~10^10^ PFU/mL) was placed on a carbon grid and stained with 2% uranyl acetate for 15 seconds. The phage samples were viewed using TEM (JEOL JEM-1011, Tokyo, Japan) at an operating voltage of 80 kV.

### Phage DNA extraction, genome sequencing, and analysis

Phage DNA was extracted using a Phage DNA isolation kit (Norgen Biotek, Thorold, Canada) following the manufacturer’s protocol. Whole-genome sequencing was performed on an Illumina NovaSeq6000 platform (Illumina Inc., San Diego, CA, USA). Whole-genome sequences were obtained using the SPAdes assembler. The whole-genome sequences were annotated using blastx and Rapid Annotations Subsystems Technology (RAST, http://rast.nmpdr.org/) (64).

### Phage receptor screening

To verify the phage receptor, transposon random mutagenesis was performed. Transposon and gentamicin-resistant gene carrying plasmid pKGL3 were cloned into *Escherichia coli* strain X7213, which is an auxotrophic mutant requiring diaminopimelic acid (DAP) (65). The bacterial cultures were prepared as follows: Four *K. pneumoniae* strains were incubated in LB broth, while *E. coli* χ7213-pKGL3 was incubated in LB broth supplemented with 20 ug/mL of gentamicin and 50 µg/mL of DAP. After overnight incubation, 1 mL of each bacterial culture was centrifuged, and the resulting pellet was resuspended and mixed with a total of 500 µL of fresh LB broth. For conjugation, the mixture was then placed onto an LB plate containing 50 µg/mL of DAP and incubated at 37°C for 6 hours. Subsequently, the bacterial cells were cultivated in fresh LB broth. To select the conjugated *K. pneumoniae*, the cultivated mixture was incubated in LB broth supplemented with 50 ug/mL of gentamicin but lacking DAP (for KPN_S3R transposon mutants, 20 ug/mL of gentamicin was used). The conjugated bacteria exhibiting phage resistance were selected using the double-layer agar method. In this method, the conjugated bacteria and high titer (~10^9^ PFU/mL) of individual phages were mixed with 0.5% LB agar. Bacterial colonies displaying both phage resistance and gentamicin resistance were isolated and subjected to sequencing analysis.

To confirm the transposon insertion site, arbitrary PCR was performed (66). Bacterial DNA was extracted using the G-spin For Bacteria Genomic DNA Extraction Kit from iNtRON Biotechnology (Seongnam, Korea), following the manufacturer’s protocol. The PCR was conducted using TransStart FastPfu Fly DNA Polymerase from TransGen Biotech (Beijing, China), with slight modifications to the manufacturer’s protocol. The first round of PCR begins with an initial denaturation step at 95°C for 5 minutes. Subsequently, cycling is performed, consisting of six repetitions. Within each cycle, denaturation occurs at 94°C for 30 seconds, followed by annealing at 30°C and elongation at 72°C for 1 minute. This cycle is then repeated 30 times, with slightly modified temperatures: denaturation at 94°C for 30 seconds, annealing at 55°C for 30 seconds, and elongation at 72°C for 1 minute. Finally, a final elongation step at 72°C is conducted for 5 minutes. The reaction is then cooled to 4°C. For the first round of PCR, one forward primer and two reverse primers were used (Forward primer: 5′-GGGAATCATTTGAAGGTTGGT-3′, reverse primer: 5′-GGCCACGCGTCGACTAGTACNNNNNNNNNNGATAT-3′ and 5′-GGCCACGCGTCGACTAGTACNNNNNNNNNNACGCC-3′). Before proceeding to the second round of PCR, the PCR products from the first round were purified using the QIAquick PCR Purification Kit (QIAGEN, Venlo, Netherlands), following the manufacturer’s protocol. In the second round of PCR, the process entails several steps. Initially, the reaction undergoes an initial denaturation at a temperature of 95°C for 5 minutes. Subsequently, cycling is carried out 30 times, with each cycle involving denaturation at 94°C for 30 seconds, annealing at 65°C for the annealing process, and elongation at 72°C for 30 seconds. Following the cycling steps, a final elongation is conducted in a single cycle at 72°C for 5 minutes. To conclude the process, the reaction is cooled down to 4°C. For the second round of PCR, each primer was used individually (Forward primer: 5′-TAGCGACGCCATCTATGTGTC-3′ and reverse primer: 5′GGCCACGCGTCGACTAGTAC-3′). Following the purification of the PCR products as described above, sequencing was performed (Macrogen, Seoul, Korea). For sequencing, the forward primer of the second round of PCR was used.

### Phage resistance suppression test

We performed a phage resistance suppression test, as previously described, with minor modifications (23). We prepared phage cocktails containing three novel phages and one previously isolated phage. Single phage was composed of a phage U2874. Cocktail 2 was composed of two phages (U2874 and phi_KPN_H2). Cocktail 3 comprised three phages (U2874, phi_KPN_H2, and phi_KPN_S3). Cocktail 4 comprised four phages (U2874, phi_KPN_H2, phi_KPN_S3, and phi_KPN_HS3). Bacteria KPN_270 inoculums (10^6^ CFU/mL) and MOI 10, 1, 0.1, 0.001, and 0.0001 of phage cocktails were placed in 96-well plates. The 96-well plates were incubated at 37°C for 24 hours, and the optical density (OD_600_) was measured every hour. The data were analyzed using VersaMax ELISA Microplate Reader (Molecular Devices, San Jose, CA, USA) and Softmax Pro (version 5.4.1, Sunnyvale, CA, USA) software. All tests were performed in five repetitions.

### Bacterial trade-off test

To investigate the trade-off effect, several aspects including bacterial growth rate, antibiotic susceptibility, biofilm formation, and virulence were tested. Briefly, for bacterial growth test involved inoculation 1 × 10^6^ CFU/mL of four different strains into individual wells of a 96-well plate. The plate was then incubated at 37°C for 24 hours. Optical density (OD_600_) measurements were taken every hour to monitor the growth of bacteria same as the lysis test.

For the antibiotic susceptibility test, we used GN4F Sensitire (Thermo Fisher, Waltham, MA, USA) and followed the manufacturer’s protocol. Briefly, we adjust bacteria McFarland standard 0.5 using DensiCHEK Plus (bioMérieux, Marcy l’Etoile, France). 10 µL of adjusted bacteria was mixed with 11 mL of Mueller Hinton (MH) broth (BD, Sparks, MD, USA). 50 µL of mixtures was loaded at GN4F plate and incubated 24 hours at 35°C. Bacterial growths were checked manually, and antibiotic susceptibility was determined according to the Clinical and Laboratory Standards Institute (CLSI) guidelines.

For biofilm assay, two methods were employed: a static culture approach that measured the biofilm formation at the bottom of the plate, and an additional measurement taken from the biofilm formed on the lids. The biofilm formation at the bottom of the plate was carried out according to the previously described protocol with minor modifications (57). Briefly, 2 µL of four strains of overnight bacterial cultures was inoculated into 200 µL of fresh Luria-Bertani (LB) broth in each well of a 96-well polystyrene plate (SPL Life Science, Pocheon, Korea). After 24 hours of static incubation at 37°C, bacteria were stained with 50 µL of 0.5% crystal violet for 20 min. The supernatant was discarded, and the plates were washed three times with distilled water. The stained dye was eluted using 99.9% of alcohol and the optical density at 550 nm (OD_550_) was determined. The biofilm formation on the lids was quantified using the Quanti-Micro Biofilm Biomass Formation Assay Kit (BIOMAX, Guri, Korea) in accordance with the manufacturer’s protocol. Briefly, the diluted microbial suspension prepared in the medium is added to the 96-well Microplate, with 200 µL per well, followed by placing the 96-peg Lid on top. The culture is then incubated at 37°C. The 96-peg Lid is removed from the cultured 96-well Microplate and washed in a saline plate, repeating this process twice. The 96-peg Lid is then transferred to a crystal violet plate and incubated for 15 minutes. The washing step is repeated twice in a saline plate. Next, the 96-peg Lid is transferred to an ethanol plate and incubated for 30 minutes. Finally, the absorbance at 590 nm is measured using a colorimetric microplate reader.

The bacterial virulence test was performed in a *Galleria mellonella* infection model. *G. mellonella* larva infection was used as described previously with minor modifications (61). *G. mellonella* larvae weighing 200–250 mg were selected randomly. Before the test, they fasted in a 90 mm petri dish in darkness at 37°C for 24 hours. Prior to injection, all individuals were swabbed with 70% ethanol. Then, 5 µL (5 × 10^6^ CFU/mL) of bacteria diluted in phosphate-buffered saline (PBS) buffer were injected into the proleg. In the control group, only PBS buffer was injected. The larvae were then incubated in the dark at 37°C in 90 mm Petri dishes. The survival of larvae was observed every 8 hours after injection for a duration of 72 hours.

The bacterial cell morphology was identified using TEM. Specimens were fixed for 12 hours in 2% glutaraldehyde-2% paraformaldehyde in 0.1M phosphate buffer (pH 7.4) and washed in 0.1M phosphate buffer, post-fixed with 1% OsO_4_ in 0.1M phosphate buffer for 2 hours and dehydrated with an ascending ethanol series (50%, 60%, 70%, 80%, 90%, 95%, 100%, and 100%) for 10 minutes each, and infiltrated with propylene oxide for 10 minutes. Specimens were embedded with a Poly/Bed 812 kit (Polysciences, Warrington, PA, USA) and polymerized in an electron microscope oven (TD-700, DOSAKA, Japan) at 65°C for 12 hours. The block is equipped with a diamond knife in the ultramicrotome and is cut into 200 nm semi-thin sections and stained toluidine blue for observation by an optical microscope. The region of interest was then cut into 80 nm thin sections using the ultramicrotome, placed on copper grids, double stained with 3% uranyl acetate for 30 minutes and 3% lead citrate for 7 minutes of staining, and imaged with a transmission electron microscopy (JEM-1011, JEOL, Tokyo, Japan) at the acceleration voltage of 80 kV equipped with a Megaview III CCD camera (Soft imaging system-Germany).

### Bacterial virulence assay in a *G. mellonella* infection model

To assess the virulence of wild-type bacteria and three phage-resistant bacteria, a *Galleria mellonella* larva infection was used as described previously (61). *G. mellonella* larvae weighing between 200 and 250 mg were randomly selected and fasted in darkness at 37°C for 24 hours prior to the test. We divided the larvae into five groups as follows: (ⅰ) control group (PBS-treated), (ⅱ) KPN_270 infection group, (ⅲ) KPN_U2874R infection group, (ⅳ) KPN_H2R infection group, and (ⅴ) KPN_S3R infection group. The four strains of bacterial inocula, each consisting of 5 × 10^6^ CFU/5 µL in PBS, were injected into the side prolegs of each larva. For the control group, 5 µL of PBS was injected. Prior to the injection, the injection larvae were swabbed with 70% ethanol. The larvae were then, incubated in the dark at 37°C in petri dishes, and the survival of larvae was determined every 8 hours for 72 hours. The larvae were considered dead when there was no movement in response to contact. All experiments were repeated three times.

### Statistical analysis

Statistical analysis was performed using GraphPad Prism 8. Significant differences among the different groups were assessed by one-way analysis of variance (ANOVA) following Tukey’s test was used to compare biofilm formation.

## Data Availability

The complete genomes of U2874, phi_KPN_S3, phi_KPN_HS3, and phi_KPN_H2 were deposited into the GenBank database under accession numbers NC_052988.1, OQ267591, OQ267592, and OQ267593, respectively.

## AFTER EPUB

*[This article was published on 29 November 2023 with Ricardo Abadie's name misspelled in the byline and errors in Table 2. The byline was corrected on 5 December 2023, and the table was corrected in the current version, posted on 22 December 2023.]*

## SUPPLEMENTAL MATERIAL

Fig. S1 legend spectrum.01258-23-s0001.docx Supplemental figure legend.

Fig. S1 spectrum.01258-23-s0002.tif Host spectrum of KPN_270_U2874R_S3R.

Table S1 spectrum.01258-23-s0003.docx List of genes that confer phage resistance.

ASM does not own the copyrights to Supplemental Material that may be linked to, or accessed through, an article. The authors have granted ASM a non-exclusive, world-wide license to publish the Supplemental Material files. Please contact the corresponding author directly for reuse.
