# Supplementary material for: Designing phage cocktails to combat the emergence of bacteriophage-resistant mutants in multidrug-resistant Klebsiella pneumoniae
Source: Microbiol Spectr. 2023 Nov 29;12(1):e01258-23. doi: 10.1128/spectrum.01258-23 (PMC10783003; doi:10.1128/spectrum.01258-23)
Supplement: Fig. S1 legend — Supplemental figure legend. [file spectrum.01258-23-s0001.docx]

**Figure S1**

Phage host spectrums against phi_KPN_S3 resistant mutant of KPN_U2874R (KPN_U2874R_S3R).
