# Supplementary figures and images for: Designing phage cocktails to combat the emergence of bacteriophage-resistant mutants in multidrug-resistant Klebsiella pneumoniae
Source: Microbiol Spectr. 2023 Nov 29;12(1):e01258-23. doi: 10.1128/spectrum.01258-23 (PMC10783003; doi:10.1128/spectrum.01258-23)

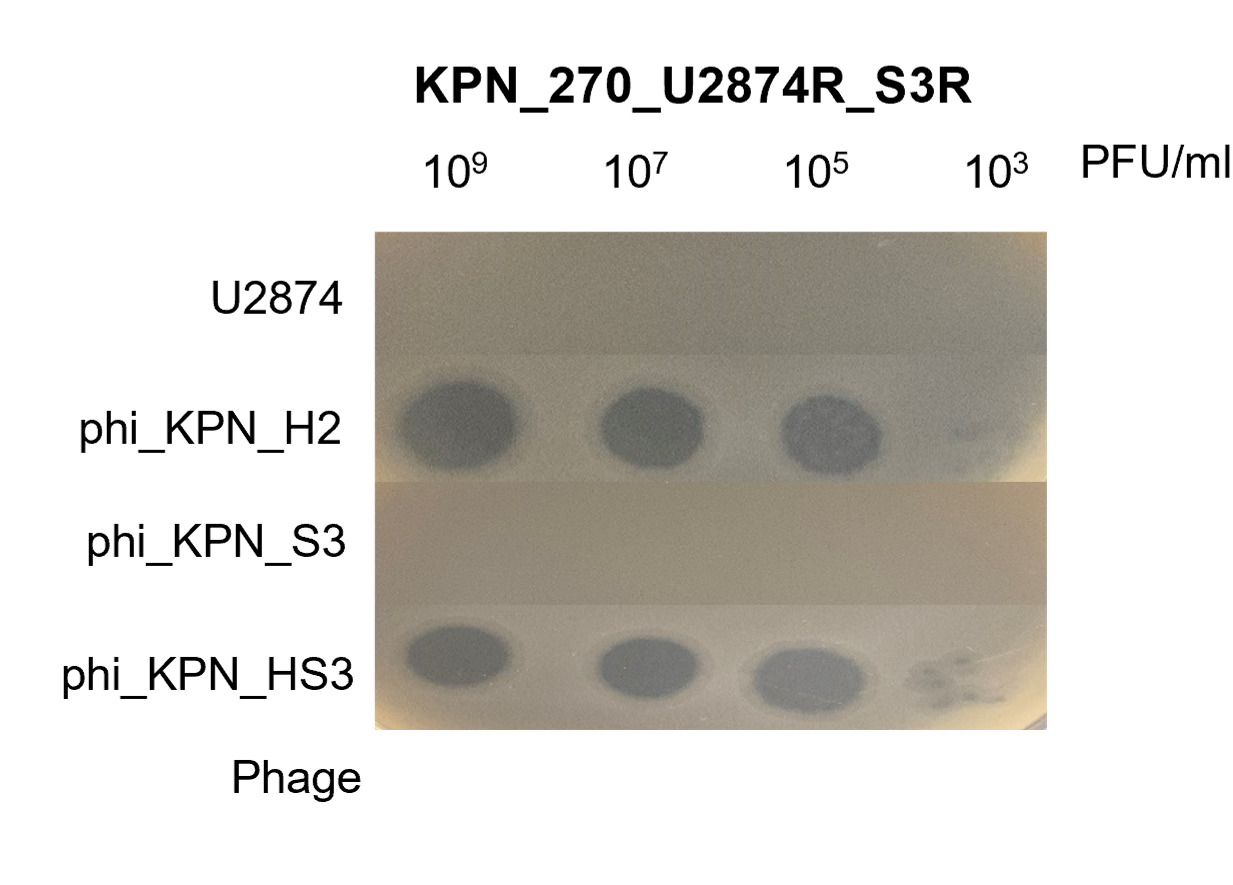

Supplement: Fig. S1 — Host spectrum of KPN_270_U2874R_S3R. [file spectrum.01258-23-s0002.tif]
