# Supplementary material for: Designing phage cocktails to combat the emergence of bacteriophage-resistant mutants in multidrug-resistant Klebsiella pneumoniae
Source: Microbiol Spectr. 2023 Nov 29;12(1):e01258-23. doi: 10.1128/spectrum.01258-23 (PMC10783003; doi:10.1128/spectrum.01258-23)
Supplement: Table S1 — List of genes that confer phage resistance. [file spectrum.01258-23-s0003.docx]

| **Bacterial strain** | **Phage** | **Mutation** | **Putative gene function** |
| --- | --- | --- | --- |
| KPN_270 | U2874 | *galE* | CPS synthesis |
|  |  | *wza* |  |
|  |  | *wzi* |  |
| KPN_U2874R | phi_KPN_H2 | *fhuA* | Ferrichrome transport complex |
|  |  | *fhuC* |  |
| KPN_H2R | phi_KPN_S3 | *galU* | LPS synthesis |
|  |  | *waaC* |  |
|  |  | *waaG* |  |
| KPN_S3R | phi_KPN_HS3 | *tonB* | Interaction with outer membrane protein |

**Supplementary Table 1** List of genes that confer phage resistance.
